# Engagement trajectories and multilevel influences on retention among adult males with advanced HIV disease in Eswatini: a mixed-methods study

**DOI:** 10.3389/fpubh.2026.1819937

**Published:** 2026-08-04

**Authors:** Fezokuhle Ncedile Khumalo, Arnold Mafukidze, Samson Haumba

**Affiliations:** 1 Georgetown University- Center for Global Health Practice and Impact, Mbabane, Eswatini; 2 Georgetown University-Center for Global Health Practice and Impact, Washington, DC, United States

**Keywords:** advanced HIV disease, disengagement, Eswatini, male engagement, mixed-methods, re-engagement, retention

## Abstract

**Introduction:**

Adult males presenting with advanced HIV disease (AHD) experience disproportionate morbidity and mortality, yet retention in care patterns among this group remain insufficiently characterized. Most studies rely on single time-point retention measures, limiting understanding of how engagement evolves over time. This study examined 6- to 12-month engagement trajectories among adult males with AHD in Eswatini and explored multilevel influences shaping retention, disengagement, and re-engagement.

**Methods:**

A mixed-methods retrospective cohort study was conducted across three high-volume HIV treatment facilities. Quantitative data were abstracted from facility AHD registers and routine clinical records for adult males recorded with AHD and documented 6 and 12-month follow-up outcomes and analyzed descriptively to characterize 6- and 12-month engagement transitions. Twelve-month outcomes were classified as active (being alive and receiving antiretroviral therapy (ART) at the study facility at 12 months, without a documented interruption exceeding 28 days) or unfavourable (death, loss to follow-up, or transfer out). Qualitative data were collected between June and September 2025 through in-depth interviews and focus group discussions with patients and healthcare workers. Thematic analysis was conducted using a socioecological framework. Quantitative and qualitative findings were integrated through joint display.

**Results:**

Among 173 participants with documented 12-month outcomes, 137 (94.5%) of men active at 6 months remained engaged at 12 months. Of the 21 men classified as lost to follow-up at 6 months, 12 (57.1%) had re-engaged by 12 months. Demographic and baseline clinical characteristics were largely not associated with 12-month outcomes. Qualitative findings identified individual (stigma, delayed acceptance), interpersonal (non-disclosure, masculinity norms), and health system factors (confidentiality concerns, tracing limitations) shaping engagement trajectories.

**Conclusion:**

Adult males with AHD exhibited distinct engagement trajectories across the first year of ART. Six-month engagement was strongly aligned with 12-month continuity, suggesting that the early treatment period may represent an important period for retention support. The observed re-engagement among over half of those classified as LTFU at 6 months indicates that interruption within the first year can be reversible. Formalizing early retention review, strengthening case-level fidelity to the AHD package, and integrating structured psychosocial assessment within routine AHD care represent actionable directions for improving long-term engagement among men.

## 1 Introduction

Advanced HIV Disease (AHD), defined as a CD4 count of less than 200 cells/μL or a WHO clinical stage 3 or 4 illness, remains a significant public health challenge globally, particularly in low- and middle-income countries (LMICs) (1). Globally, a substantial proportion of people living with HIV continue to present with AHD, contributing significantly to HIV-related morbidity and mortality (2). Sub-Saharan Africa bears the brunt of the global HIV burden, accounting for 67% of all people living with the virus (3, 4). The prevalence of AHD among individuals initiating antiretroviral therapy (ART) remains high, with estimates ranging from 32 to 71% (5, 6). A systematic review of the prevalence of AHD across Sub-Saharan Africa estimated a pooled prevalence of 58.7% (7). Preliminary findings from community-based Population HIV Impact Assessment (PHIA) surveys conducted between 2016 and 2018 across nine countries revealed that 11–22% of newly diagnosed adults had a CD4 cell count below 200 cells/μL, highlighting the ongoing challenge of late presentation to care (8). To this end, the World Health Organization has established guidelines for the management and prevention of AHD, emphasizing the importance of early detection, prompt initiation of ART, and appropriate prophylactic treatments to manage and reduce associated complications such as opportunistic infections (9).

In Eswatini, a country that has made remarkable strides in HIV epidemic control, the implementation of the Test and Start strategy for ART [where all individuals diagnosed with HIV are immediately initiated on ART regardless of CD4 count or WHO staging (10)] has contributed to achieving the UNAIDS 95-95-95 goals in 2020 (11). However, despite the achievement of HIV epidemic control, a disproportionately high number of individuals still present with AHD, indicating gaps in early HIV detection, retention in care, and effective ART adherence (11). Eswatini has high ART coverage (97.3% of diagnosed individuals), and yet, 12.6% still present with AHD at ART initiation (11). Experts in the AHD field have called for expansion of global targets beyond the current UNAIDS 95-95-95 cascade, to specifically address the complex management of AHD. Specifically, there is ongoing advocacy for a revised and complementary AHD-specific “95-95-95 cascade,” encompassing (1) universal and timely CD4 testing for all individuals newly initiating ART, (2) comprehensive screening for opportunistic infections using TB-LAM and CrAg-LFA tests among patients identified with CD4 counts below 200 cells/μL, and (3) immediate initiation of treatment for those testing positive for either TB or cryptococcal infection (12). Furthermore, there remains an urgent need for improved monitoring and reporting of HIV-related mortality, particularly within populations experiencing AHD, to accurately assess programmatic effectiveness and address continuing disparities.

The HIV care cascade outlines the sequential steps required for optimal management of HIV, from diagnosis through linkage to care, retention in treatment, adherence to ART, and ultimately, achieving sustained viral suppression (13). However, substantial barriers exist at each stage of this cascade, particularly among adult males. Delayed HIV diagnosis, suboptimal linkage to care post-diagnosis, inadequate retention in care, inconsistent adherence, and challenges in achieving long-term viral suppression remain pervasive issues (14). Evidence indicates that adult males experience distinct and pronounced challenges at these critical stages, often related to structural, social, and individual-level factors, which contribute to their disproportionately poorer retention and viral suppression outcomes (14, 15). In Eswatini specifically, adult males exhibit lower retention rates compared to females, despite similar access to healthcare services, highlighting a critical gender disparity requiring targeted strategies to address effectively (16).

In this study, engagement trajectories refer to transitions in care status between 6 and 12 months following ART initiation. Loss to follow-up (LTFU) refers to an individual on HIV treatment missing their medical appointments or stopping their medication for over 28 days (17). Re-engagement in care is the subsequent return to care after previously being lost to follow-up. Consequently, being active in care in this study refers to being alive and receiving ART at the study facility at 12 months, without a documented interruption exceeding 28 days. High rates of disengagement among adult males present significant public health concerns, as poor retention undermines individual health outcomes, contributes to increased morbidity and mortality, and increases the risk of onward HIV transmission due to unsuppressed viral loads (18). Addressing these critical challenges is essential for sustained HIV epidemic control and for achieving the global objective of ending HIV/AIDS as a public health threat by 2030.

A cornerstone of effective HIV treatment, retention in care means that PLHIV are more likely to adhere to ART and can ultimately achieve sustained viral suppression (19). Consistent retention improves individual health outcomes by reducing HIV-related morbidity and mortality, and plays a crucial role in preventing onward transmission at a population level. The third 95 of the 95–95-95 goals emphasizes achieving viral suppression among all PLHIV, a goal that is only possible if individuals remain engaged in long-term HIV care and consistently adhere to ART (20). Although ART initiation is a critical milestone, long-term retention remains fragile in many high-burden settings. Disengagement from care is rarely the result of a single factor; rather, it reflects the cumulative impact of structural constraints (e.g., transport costs, long travel distances), social dynamics (e.g., stigma and limited disclosure), and individual-level vulnerabilities, including psychological distress and competing livelihood demands (21–24). These intersecting pressures can lead to treatment interruption, disease progression, and increased risk of onward transmission. Sustained engagement therefore depends not only on successful linkage, but on health systems that anticipate and mitigate these pressures across the care continuum. Importantly, these barriers are not experienced uniformly. Adult males are disproportionately affected by sociocultural expectations around masculinity, employment-related mobility, and lower health-seeking behaviour, which together may increase vulnerability to disengagement (24, 25).

In Eswatini, retention among men with AHD has largely been described using single time-point indicators, with limited exploration of how engagement transitions unfold over time. Although men are known to delay testing, initiate ART late, and disengage more frequently than women, few studies in this context integrate longitudinal engagement transitions with qualitative examination of the multilevel determinants shaping these patterns in this sub-population group (16, 25, 26). In response to this gap in the literature, the present mixed-methods study aims to characterize care trajectories among adult males with AHD and to examine the decision points shaping retention, disengagement, and re-engagement in HIV care in Eswatini. A mixed-methods approach is particularly suited to integrating routine clinical outcome data with lived experiences, allowing for a more comprehensive understanding of how individual, clinical, and health system factors interact to influence care continuity (27, 28). The study therefore aims to explore the following research question: How do adult males with AHD in Eswatini experience and navigate retention, disengagement, and re-engagement in HIV care?

The present study applied a socio-ecological framework to comprehensively explore the diverse factors affecting retention among adult males diagnosed with AHD. According to this framework, determinants of health-related behaviours and outcomes occur across multiple levels: individual (patient), healthcare system, community, and broader societal structures (29).

## 2 Methods

### 2.1 Study design and setting

The study employed a mixed-methods, retrospective cohort design to examine the engagement trajectory of adult males with AHD in Eswatini. Quantitative analysis of patient records and qualitative interviews with both patients and healthcare workers were conducted. The study was conducted at three purposively selected health facilities that provide comprehensive HIV care and treatment services, with a focus on managing patients with AHD. Facilities were selected based on patient volume, service availability, and documented challenges in retention and re-engagement of men in HIV care. Specifically, site selection was guided by the following criteria: 1) High CD4 testing coverage (≥80% of new ART initiators having a documented baseline CD4 result) among new ART clients; 2) Highest number of new ART clients; 3) Highest number of AHD cases among new ART clients; and 4) True high-volume facilities (based on programmatic data showing clinics with the highest absolute numbers of new ART initiations and baseline CD4 tests documented in EHR during the study period). These facilities provide integrated HIV services, including HIV testing and counselling, ART initiation and refill services, management of ART-related complications, AHD screening and management (CD4 testing, TB screening, cryptococcal antigen testing), PMTCT, PrEP and PEP, and integrated HIV and non-communicable disease care. Electronic patient records from the national electronic health records (EHR) were used to map patient trajectories and identify critical gaps in retention and re-engagement.

### 2.2 Study population

This retrospective cohort analysis assessed retention in HIV care and re-engagement among adult males (aged 18+) with AHD receiving care at three high-volume health facilities in Eswatini: AHF Matsapha Clinic, AHF LaMvelase Clinic, and Mankayane Government Hospital. The study included adult males aged 18 years or older who were recorded in the facility AHD registers during the AHD register abstraction period from October 2023 to March 2025. AHD classification was based on documented baseline CD4 < 200 cells/μL and/or WHO clinical stage 3 or 4 illness. For follow-up assessment, the index date was defined as the baseline CD4 test date recorded in the AHD register. Six and 12-month outcomes were assessed relative to this baseline CD4 test date. Across the three sites, CD4 testing coverage among newly initiated ART clients ranged from 86 to 96%. Among those tested, the proportion with CD4 < 200 cells/μL ranged from 36 to 56%.

### 2.3 Sample size considerations and sampling strategy

The study initially included all eligible AHD cases diagnosed between October 2023 and September 2024 (*n* = 123). Preliminary power analysis indicated this sample was insufficient to achieve 80% power to detect a 20% absolute difference in 12-month outcomes at a 5% significance level, accounting for clustering by facility (ICC = 0.02) (30). Power calculations conducted in Stata 17 (StataCorp, College Station, TX, USA) estimated approximately 60% power with the available sample. The study period was therefore extended to March 2025. Based on historical enrollment of 10–12 new AHD cases per month, the extension was expected to yield approximately 180 participants, exceeding the required minimum sample size of 154 and achieving ≥80% power.

For the qualitative component, purposive sampling was used to capture variation in clinical severity, care status, and service delivery experience among adult males with AHD and healthcare workers involved in HIV/AHD care. Routine AHD register and clinical record data were used to construct a sampling frame for male patient participants. Two derived variables were used to guide stratification: AHD severity (severe AHD, moderate AHD, or post-AHD recovery), based on CD4 count and opportunistic infection indicators, and care status (retained, interrupted in treatment, or re-engaged), based on documented treatment continuity. A 3 × 3 matrix mapping severity against care status was developed to ensure representation across key trajectory groups. Participants selected for IDIs were not included in FGDs to avoid overlap.

Qualitative data were collected from 27 participants, comprising 16 male PLHIV/AHD participants and 11 healthcare workers. Among male participants, 9 participated in IDIs and 7 participated in FGDs. Healthcare workers were purposively recruited from participating facilities based on their involvement in HIV/AHD service delivery, including ART initiation, AHD screening and management, patient tracing, pharmacy services, adherence counselling, and psychosocial support. Healthcare worker participants included doctors, nurses, pharmacy personnel, and adherence officers. Participation was voluntary, and all healthcare workers provided informed consent before taking part in interviews. Data were collected at two participating facilities, selected based on feasibility and participant availability during the qualitative data collection period.

In addition, one FGD was conducted with female PLHIV to provide contextual insight into gendered experiences of HIV care and to support interpretation of gender-related barriers and facilitators. Data from the female FGD were used comparatively and contextually, rather than as primary evidence of male engagement trajectories. Participation in the qualitative component was voluntary, and all participants provided informed consent before taking part in interviews or focus group discussions. Sampling continued until recurring themes were observed across participant accounts and no substantially new themes were identified within the main domains of inquiry.

### 2.4 Inclusion and exclusion criteria

Quantitative data inclusion criteria included adult males aged 18 years or older who were recorded in the facility AHD register, had documented AHD based on CD4 < 200 cells/μL and/or WHO clinical stage 3 or 4 illness, received HIV care at one of the three selected facilities, and had sufficient routine record documentation to classify 6 and 12-month engagement outcomes.

Participants were excluded if clinical records were incomplete or missing data required to classify AHD or retention status, if diagnosis occurred outside the defined study period, or if they transferred into the study facilities after ART initiation and therefore lacked sufficient baseline information at the study site. Documented transfer-out during follow-up was retained as a programme outcome and reported separately within the 12-month outcome classification.

For the qualitative component, healthcare workers were eligible if they were employed at one of the participating facilities and directly involved in HIV/AHD service delivery. Eligible cadres included doctors, nurses, pharmacists or pharmacy technicians, adherence counsellors, psychosocial officers, and expert patients. Doctors were required to be registered with the Eswatini Medical and Dental Council, have at least 2 years of clinical practice experience, and be directly involved in ART initiation, AHD management, or HIV-related clinical decision-making. Nurses were required to be registered with the Eswatini Nursing Council, have at least 1 year of practice experience, and be involved in routine ART services, patient monitoring, or AHD care. Pharmacists and pharmacy technicians were required to be registered with the relevant professional council, have at least 1 year of practice experience, and be involved in dispensing ART or AHD-related medications. Adherence counsellors, psychosocial officers, and expert patients were eligible if they provided adherence counselling, psychosocial support, telephone follow-up, or home visits for patients who had interrupted treatment. All healthcare workers were required to provide informed consent before participation.

### 2.5 Data collection

Quantitative data were extracted from facility AHD registers and routine clinical records in May 2025 and analysed using Stata version 17 (StataCorp, College Station, TX, USA). The AHD register included follow-up fields for outcome status at 6 and 12 months after the baseline CD4 test. These fields were used to classify engagement status at each time point and were verified against routine clinical records where available. Extracted variables included demographic characteristics (age, marital status), anthropometrics (baseline weight, BMI) clinical indicators (CD4 count, WHO stage, opportunistic infections), 6-month care status, and 12-month outcomes. Outcome data were obtained through routine documentation of retention, loss to follow-up, transfer, or death within the EHR. Qualitative data were collected through semi-structured IDIs and FGDs conducted between June and September 2025. Interview guides were developed to explore participants’ experiences of HIV diagnosis, treatment initiation, retention, interruption, and re-engagement in care. Questions were informed by the study objectives and the socioecological framework guiding the broader analysis. IDIs were conducted to capture individual care trajectories in depth, as well as HCW perspectives. FGDs were used to explore shared experiences, norms, and perceived structural influences on retention. Healthcare workers participated in key-informant interviews rather than FGDs. These interviews explored service delivery processes, implementation of the AHD package, ART initiation and follow-up, retention and tracing systems, confidentiality concerns, clinic workflow, and perceived barriers and facilitators to re-engagement among men with AHD. Interviews were conducted in siSwati or English, according to participant preference, in private spaces within the health facilities to ensure confidentiality. All interviews were audio-recorded with participant consent and supplemented with field notes documenting contextual observations and reflections. Interviews lasted approximately 40 min. Audio recordings were transcribed verbatim and translated into English prior to analysis.

### 2.6 Data analysis

Quantitative analyses were restricted to participants with documented 12-month outcomes and sufficient potential follow-up from the baseline CD4 test date to the May 2025 data extraction date. Descriptive statistics were used to summarize baseline demographic and clinical characteristics. Continuous variables were assessed for distribution using summary statistics and visual inspection of distributional parameters. Normally distributed variables were reported as means and standard deviations (SD), while skewed variables were summarized using medians and interquartile ranges (IQR). Categorical variables were summarized using frequencies and percentages. The primary outcome was 12-month retention in care status, dichotomized as active in care versus unfavourable. Active in care was defined as being alive and receiving ART at the study facility at 12 months without a documented interruption exceeding 28 days. The unfavourable category included death, loss to follow-up, or documented transfer-out by 12 months. This composite was used to describe absence from active care at the original study facility from a programme monitoring perspective. Because death, LTFU, and transfer-out represent distinct clinical and programmatic events, the components of the composite outcome were also reported separately and interpreted cautiously. Death was treated as a terminal outcome in the descriptive pathway analysis. Once death occurred, no further transitions in engagement status were possible within the analytic framework. The study did not employ formal time-to-event or competing risks modelling; analyses were based on status classification at predefined time points (6 and 12 months). Baseline characteristics were compared between outcome groups. For continuous variables, independent sample t-tests were used for normally distributed variables and Wilcoxon rank-sum tests were used for non-normally distributed variables. For categorical variables, Pearson’s chi-square tests were applied; Fisher’s exact tests were used where expected cell counts were small. To describe care trajectory over time, 6-month retention status was cross tabulated with 12-month outcomes to examine transitions in care status. This analysis was descriptive and intended to illustrate patient pathways rather than to infer causality. A two-sided *p*-value of <0.05 was considered statistically significant.

Qualitative data were analysed using inductive thematic analysis. Transcripts were read repeatedly to achieve familiarization, after which initial codes were generated and iteratively grouped into broader themes through constant comparative analysis across interviews and focus groups. Themes were reviewed and refined to ensure alignment with the socioecological framework guiding the study. Quantitative and qualitative findings were subsequently integrated using a joint display approach to compare care trajectories with participants’ reported experiences. Quantitative patterns in retention, interruption, and re-engagement were examined alongside qualitative themes to better understand how and why transitions in care occurred.

## 3 Results

### 3.1 Baseline characteristics by 12-month outcome

A total of 173 adult males with AHD were included in the analysis, of whom 149 (86.1%) were active in care at 12 months and 24 (13.9%) had an unfavourable outcome (Table 1). Of the 24 unfavourable 12-month outcomes, 6 (25.0%) were deaths, 17 (70.8%) were loss to follow-up, and 1 (4.2%) was documented transfer-out. The mean age of participants was 42.3 ± 8.5 years and did not differ between outcome groups (*p* = 0.79). Most participants were single (69.4%), followed by married/cohabiting (26.6%).

**Table 1:** Baseline characteristics of adult males with AHD by 12 month outcome status.

Characteristic	Outcome at 12 Months		Total (*n* = 173)	*p*-value
	Active (*n* = 149)	Unfavourable (*n* = 24)		
	*n* (%)	*n* (%)	*n* (%)	
Demographics				
Age, years (mean ± SD)	42.2 ± 8.4	42.7 ± 9.2	42.3 ± 8.5	0.79
Marital status				0.52
Single	104 (86.7)	16 (13.3)	120 (69.4)	
Married/cohabiting	40 (87.0)	6 (13.0)	46 (26.6)	
Widowed/divorced/separated	5 (71.4)	2 (28.6)	7 (4.0)	
Anthropometrics				
Baseline weight, kg (median [IQR])	65 [58.9–75.6]	68 [61.0–76.0]	65 [59.0–76.0]	0.82
Baseline BMI (kg/m^2^, mean ± SD)	23.3 ± 4.3	23.1 ± 3.6	23.3 ± 4.2	0.84
Baseline CD4 (cells/μL, median [IQR])	85 [45–119]	144 [45.5–162.5]	90 [47–127]	0.09
AHD clinical indicators				
WHO stage 3/4 OI				0.26
Yes, *n* (%)	28 (93.3)	2 (6.7)	30 (17.3)	
No, *n* (%)	121 (84.6)	22 (15.4)	143 (82.7)	
TB presumptive, *n* (%)				0.71
Yes, *n* (%)	131 (86.2)	21 (13.8)	152 (89.9)	
No, *n* (%)	14 (82.4)	3 (17.6)	17 (10.1)	
CrAg screened, *n* (%)				0.014
Yes, *n* (%)	145 (87.9)	20 (12.1)	165 (95.4)	
No, *n* (%)	4 (50.0)	4 (50.0)	8 (4.6)	

1. Unfavourable outcome includes death, loss to follow-up, or transfer out at 12 months.

2. Continuous variables are presented as mean ± SD or median [IQR] as appropriate.

3. TB presumptive status was missing for 4 participants, all in the active outcome group. Percentages for this variable are calculated using available data (*n* = 169).

Median baseline weight was 65 kg [IQR 59.0–76.0], and mean baseline BMI was 23.3 ± 4.2 kg/m (2). There were no significant differences in baseline weight (*p* = 0.82) or BMI (*p* = 0.84) by outcome status. The overall median baseline CD4 count was 90 cells/μL [IQR 47–127]. Although participants with unfavourable outcomes had a higher median CD4 count (144 cells/μL) compared to those retained (85 cells/μL), this difference did not reach statistical significance (*p* = 0.09). Regarding AHD clinical indicators, 17.3% of participants had a documented WHO stage 3 or 4 opportunistic infection at baseline, with no significant difference by outcome (*p* = 0.26). Among participants with documented WHO stage 3/4 conditions (*n* = 30), confirmed tuberculosis (TB) was most common (*n* = 18; 60.0%). Other reported conditions included chronic diarrhoea (*n* = 5; 16.7%), severe weight loss (*n* = 2; 6.7%), cryptococcal infection (*n* = 1; 3.3%), Pneumocystis pneumonia (*n* = 1; 3.3%), oral malignancy (*n* = 1; 3.3%), renal failure (*n* = 1; 3.3%), and clinically documented stage IV disease without a specified opportunistic infection (*n* = 1; 3.3%). TB presumptive status was also not associated with 12-month outcome (*p* = 0.71). However, cryptococcal antigen (CrAg) screening status was significantly associated with outcome (*p* = 0.014).

### 3.2 Engagement trajectories from 6 to 12 months

Six-month retention status was cross-tabulated with 12-month outcomes to examine engagement transitions over time (Table 2). Among participants who were active at 6 months (*n* = 145), 137 (94.5%) remained active at 12 months, while 8 (5.5%) experienced an unfavourable outcome. Of the 21 participants classified as lost to follow-up (LTFU) at 6 months, 12 (57.1%) had re-engaged in care by 12 months, whereas 9 (42.9%) remained unfavourable. All participants recorded as dead (*n* = 6) or transferred out (*n* = 1) at 6 months remained in the unfavourable category at 12 months.

**Table 2:** Six-month retention status and corresponding 12-month outcomes among adult males with AHD.

Six month outcome status	Active at 12 months, *n* (%)*	Unfavourable at 12 months, *n* (%)	Total (*n*)
Active	137 (94.5)	8 (5.5)	145
Dead	0 (0.0)	6 (100.0)	6
LTFU	12 (57.1)	9 (42.9)	21
Transferred out	0 (0.0)	1 (100.0)	1
Total	149	24	173

*Percentages represent row proportions. Unfavourable outcome includes death, loss to follow-up, or transfer out at 12 months.

**Figure 1:**
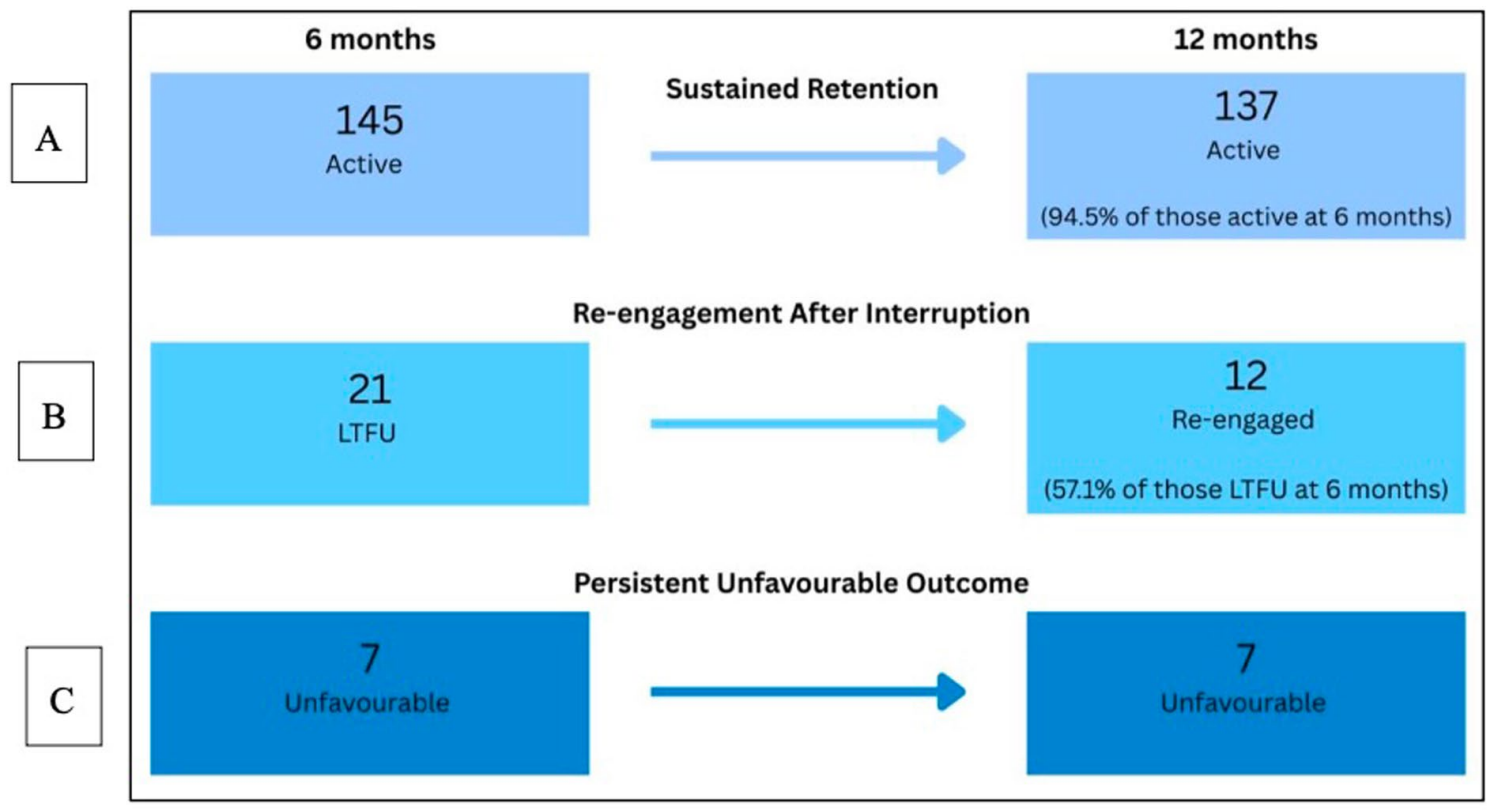
Engagement trajectories from 6 to 12 months among adult males with advanced HIV disease in Eswatini. **(A)** Sustained retention. **(B)** Re-engagement after interruption. **(C)** Persistent unfavourable outcome. Percentages represent row proportions. Unfavourable outcomes include death, loss to follow-up, or transfer-out at 12 months. Of the 145 participants active at 6 months, 137 remained active and 8 had an unfavourable outcome by 12 months. Of the 21 participants classified as LTFU at 6 months, 12 re-engaged and 9 remained unfavourable at 12 months. All participants classified as dead (*n* =6) or transferred out (*n* = 1) at 6 months remained in the unfavourable category at 12 months. Overall, the 24 12-month unfavourable outcomes comprised 6 deaths, 17 LTFU, and 1 documented transfer-out.

Overall, three dominant trajectories were observed: sustained retention among those active at 6 months, partial re-engagement among those previously LTFU, and persistence of unfavourable outcomes among those already classified as dead or transferred (Figure 1).

### 3.3 Multilevel factors influencing retention and re-engagement

Qualitative findings are presented according to socioecological levels to illustrate how individual, interpersonal, community, and health system factors influenced engagement trajectories among adult males with AHD.

#### 3.3.1 Individual level factors

At the individual level, stigma, low self-esteem, and delayed acceptance of diagnosis were prominent barriers to sustained engagement. Participants described fear of being recognized at HIV clinics and discomfort with visible ART documentation.

*“It starts when we are diagnosed, the ART book has stigma. We see our friends, we do not want to meet there.”* (FGD, MLHIV).

Men also described difficulty accepting their HIV status, which delayed linkage and re-engagement.

*“Males do not like hospitals. Low self-esteem… not accepting easily.”* (FGD, MLHIV).

Conversely, personal motivation, family support, and gradual acceptance of HIV status emerged as facilitators of adherence and continued care. Male participants described engagement as improving when they received encouragement, understood the consequences of stopping treatment, and became more comfortable with lifelong ART.

*“I have received support from my family since I struggled with starting treatment when I discovered I was HIV positive… Being told that there are people who stopped taking this treatment and fell sick and died, that pushed me to taking my medication properly.”* (FGD, MLHIV).

#### 3.3.2 Interpersonal and household factors

Non-disclosure within families created ongoing anxiety around medication-taking and clinic attendance. Participants described navigating treatment secretly to avoid household tension.

*“An observant family… especially not known from the family!”* (FGD, MLHIV).

Peer networks could function both as pressure and support. Some men reported being encouraged by friends to seek care when visibly unwell.

*“They joke around when you get thin. They tell you to go get checked.”* (FGD, MLHIV).

Supportive relationships with healthcare providers also facilitated retention. Participants valued continuity and respectful communication.

*“The nurses here are very friendly… they allow you to communicate openly.”* (FGD, MLHIV).

#### 3.3.3 Community level factors

Employment obligations were described as a structural barrier to clinic attendance and medication continuity. Male participants described work-related constraints not only in terms of time, but also in relation to privacy, repeated clinic attendance, and fear that colleagues might infer their HIV status.

*“Well, I think work does have an effect because people are now smart and they can predict your schedules… Then they would assume that maybe you are on ART.”* (FGD, MLHIV).

*“Having to go with my ART pills to work is a problem.”* (FGD, MLHIV).

Food insecurity also affected adherence, particularly when ART was taken on an empty stomach.

*“Once you take the pill on an empty stomach you can just feel yourself getting sick.”* (FGD, MLHIV).

#### 3.3.4 Health system level factors

At the health system level, participants and healthcare workers described several constraints affecting sustained retention and re-engagement.

Staff shortages limited active tracing and follow-up of patients who disengaged from care.

*“We have less number of staff members that call the clients. We cannot reach all the clients.”* (KII, ART Nurse).

Transportation challenges reduced the feasibility of home visits for patients who missed appointments.

*“Lack of transport for home visits.”* (KII, Expert Client).

Concerns regarding privacy and unintended status disclosure related to clinic layout were also raised, as participants described structural features of clinic layout that increased perceived risk of unintended HIV status disclosure.

*“Confidentiality part is not up to standard… ART dispensary should be in the ART department.”* (KII, Pharmacy Technician).

Despite these challenges, re-engagement efforts such as “welcome back” campaigns were described as supportive, though limited by resource constraints.

### 3.4 Integration of quantitative and qualitative insights

Quantitative and qualitative findings were integrated using a joint display approach to examine how observed care trajectories aligned with participants’ reported experiences (31, 32) (Table 3). Patterns of sustained retention, interruption in treatment, and re-engagement at 12 months were interpreted alongside multilevel determinants identified in interviews and focus groups. Sustained retention was associated with individual acceptance and supportive provider relationships. Re-engagement following interruption was often triggered by social influence or illness severity. Persistent disengagement was linked to structural and health system barriers, including stigma, work-related constraints, and limited tracing capacity.

**Table 3:** Joint display of engagement trajectories and socioecological determinants of retention and re-engagement.

Care trajectory	Quantitative pattern	Qualitative explanation
Sustained retention (Active ➔ Active)	137/145 (94.5%)	Acceptance of diagnosis Responsibility toward children Positive provider relationships Clinic-based support
Re-engagement (LTFU ➔ Active)	12/21 (57.1%)	Peer encouragement “Welcome back” initiatives Return after severe illness Family influence
Persistent unfavourable (LTFU ➔ Unfavourable)	9/21 (42.9%)	Work-related mobility Non-disclosure at home Stigma at clinics Staff shortages for tracing Transport barriers

## 4 Discussion

This mixed-methods study characterized 6- to 12-month engagement trajectories among adult males presenting with AHD in Eswatini and examined the multilevel determinants shaping retention, disengagement, and re-engagement. The study moved beyond a static cross-sectional view of retention estimates to highlight the dynamic nature of care engagement among men with AHD. These findings align with literature that emphasizes the cyclical nature of HIV care engagement, with clients moving in and out of care over time (33–35).

Three dominant trajectories were observed including sustained retention among those active at 6 months, partial re-engagement among men previously lost to follow-up, and persistence of unfavourable outcomes among a subset of disengaged individuals. Notably, 94.5% of men active at 6 months remained engaged at 12 months, suggesting that early stabilization in care represents a critical window for long-term retention. At the same time, over half of those classified as LTFU at 6 months had re-engaged by 12 months, reinforcing that disengagement among men is often fluid rather than permanent. These patterns are consistent with evidence from Sub-Saharan Africa that demonstrates a higher risk of attrition within early months following ART initiation, with engagement stabilizing thereafter (36, 37). Tracing and cohort studies further show that a substantial proportion of patients recorded as LTFU subsequently return to care or are found to be alive, underscoring the dynamic nature of engagement (38, 39). The 6-month time point may therefore represent a programmatically important juncture for targeted retention support among adult men with AHD (Table 4).

**Table 4:** Study findings and corresponding programmatic recommendations.

Key empirical finding	Programmatic recommendation
Six-month engagement status was strongly aligned with 12-month outcomes; most men active at 6 months remained active at 12 months.	*Operationalize the first 9 months as a structured stabilization phase within AHD care.* While guidelines define routine follow-up, facilities may incorporate a formalized 6-month review to assess adherence stability, refill continuity, and emerging structural barriers, with timely adjustment of DSD intensity where appropriate.
A substantial proportion of men classified as LTFU at 6 months had re-engaged by 12 months, indicating fluid movement between engagement states.	*Strengthen early re-engagement intensity for newly interrupted clients during the first year of ART.* Within existing IIT monitoring systems, facilities may emphasize rapid follow-up and low-barrier return pathways before prolonged disengagement occurs, recognizing interruption as part of a dynamic care trajectory rather than a terminal state.
A small number of participants lacked documented CrAg screening, and unfavourable outcomes were more frequent in this group.	*Strengthen timely completion and documentation of the WHO-recommended AHD package.* In addition to aggregate reporting, facilities may benefit from routine verification that each AHD client has completed all recommended components of the package, using simple checklist or EHR prompts to reduce missed steps.
Qualitative findings highlighted stigma, delayed disclosure, masculinity norms, employment pressures, and confidentiality concerns influencing continuity of care.	*Embed structured early psychosocial stabilization within routine AHD management.* A brief, standardized counselling module within the first month of ART, addressing disclosure planning, mental health coping, work-related adherence challenges, and confidentiality reassurance, may support early stabilisation without creating parallel mental health services.

Baseline clinical and demographic characteristics were largely not associated with 12-month outcomes, underscoring that biomedical severity alone did not explain retention patterns in this population. Notably, the non-significant direction of some baseline comparisons ran counter to clinical expectation as participants with unfavourable outcomes had a higher median CD4 count, and those with documented WHO stage 3/4 opportunistic infections were less likely to have an unfavourable outcome. This pattern may reflect the programmatic nature of the composite outcome, which primarily captured LTFU and death, rather than clinical deterioration alone. It may also suggest that men with more clinically apparent AHD received more intensive clinical attention or follow-up, while those with less advanced measured disease may have been more vulnerable to mobility-related interruption, silent disengagement, or weaker tracing visibility. The observed association between CrAg screening status and 12-month outcome should be interpreted cautiously. Although participants without documented CrAg screening had a higher proportion of unfavourable outcomes, this pattern was based on a small number of unscreened participants (*n* = 8), of whom four had unfavourable outcomes. The direction of causality cannot be established in this descriptive analysis. It is possible that some participants were not screened because they disengaged, transferred, or clinically deteriorated before screening could be completed, rather than lack of screening directly contributing to unfavourable outcomes. Nevertheless, the finding may point to the importance of timely completion and documentation of the AHD package in routine care. The influence of gender norms, livelihood constraints, health system responsiveness, and service delivery models on sustained engagement in HIV care is well researched (40–43). The WHO guidelines for managing advanced HIV disease emphasize systematic screening for cryptococcal infection among individuals with CD4 < 200 cells/μL as part of a comprehensive mortality-reduction strategy (9, 44). Evaluations of AHD package rollout in routine care settings have demonstrated variability in CrAg screening coverage, suggesting persistent implementation challenges despite clear clinical guidance (45–47). While causal inference cannot be drawn from these descriptive findings, the observed pattern underscores the importance of full implementation fidelity of the AHD package in high-risk populations, such as adult males with AHD.

Qualitative findings contextualized these trajectories within a socioecological framework. At the individual level, stigma, delayed acceptance of diagnosis, and low self-esteem influenced early disengagement. While “stigma” was frequently invoked in participant narratives, it appeared to encompass multiple distinct dimensions with different implications for care engagement. For some men, stigma reflected fear of social rejection or community gossip; for others, it centred on anticipated relationship consequences, including loss of intimate partners or family support. Economic vulnerability also emerged implicitly, as disclosure could threaten employment stability or social standing. In addition, several accounts reflected internalized shame and perceived moral judgment, while others pointed to masculinity-based expectations that equate illness with weakness. These differentiated expressions of stigma suggest that disengagement is shaped not by a single abstract barrier, but by layered social risks that intersect with identity, livelihood, and relational security. These findings are consistent with previous scholars that identify internalized stigma and psychological adjustment to diagnosis as key drivers of delayed linkage and treatment interruption among men (48–50). However, while much of the literature emphasizes stigma primarily at the point of testing or initial linkage, our findings suggest that its influence may persist beyond ART initiation, shaping early retention among men presenting with advanced disease. At the interpersonal and community levels, non-disclosure, employment demands, and social expectations of masculinity shaped care-seeking behaviour (51, 52). At the health system level, confidentiality concerns, limited tracing capacity, and resource constraints affected the likelihood of re-engagement. Similar structural barriers have been documented in evaluations of ART service delivery models in sub-Saharan Africa, where clinic congestion, fear of inadvertent disclosure, and weak follow-up systems contribute to disengagement (53–56). These multilevel determinants reinforce that retention among men with AHD cannot be understood solely as an issue of adherence but must be treated and approached as a systems-level challenge.

This study adds to the existing literature in several ways. A key strength of this study is the use of longitudinal routinely collected data to examine engagement at two time points within the first year of ART, providing a more nuanced understanding of continuity and re-engagement patterns. This study cross-tabulated 6 and 12-month outcomes, exploring engagement as movement across states rather than a binary classification (retained vs. interrupted in treatment, offering a more nuanced understanding of male retention patterns within routine program data. Second, the mixed-methods design strengthens interpretability. Quantitative data identified trajectory patterns, while qualitative data provided explanatory depth at specific decision points. Third, the socioecological framing allows translation of findings into actionable implementation insights. Rather than attributing disengagement solely to individual behaviour, the study demonstrates how structural constraints such as employment mobility and limited tracing infrastructure interact with stigma and gender norms to shape care continuity.

Several limitations warrant consideration. As a retrospective cohort analysis using routine clinical data, outcome misclassification and incomplete documentation may have occurred. In particular, some care transitions, such as undocumented transfer or re-engagement at another facility, may not have been fully captured. The composite unfavourable outcome combined death, LTFU, and transfer-out, which represent distinct clinical and programmatic events. Although transfer-out accounted for only one unfavourable outcome, documented transfer may reflect mobility or continuation of care elsewhere rather than poor clinical outcome. The study was conducted in high-volume facilities and may not reflect retention patterns in lower-volume or rural settings. Additionally, the analysis was descriptive and does not establish causal relationships. This is particularly relevant for the observed CrAg screening pattern, which was based on a small number of unscreened participants and may be affected by reverse causation. However, the integration of qualitative findings mitigates interpretive limitations by providing explanatory context for observed transitions.

## 5 Conclusion and recommendations

In conclusion, adult males presenting with advanced HIV disease in Eswatini demonstrate heterogeneous, yet interpretable engagement trajectories shaped by interacting socioecological influences. Early engagement appeared to mark an important period for retention support, with 6-month status closely aligned with subsequent continuity, while disengagement was often reversible rather than permanent. These findings highlight that retention among men with AHD reflects dynamic interactions between behavioural, structural, and health system factors.

The findings of this study suggest that improving outcomes among men presenting with advanced HIV disease will require attention to how engagement unfolds over time, rather than solely to whether clients are retained at a single endpoint. The observed alignment between 6-month and 12-month engagement suggests that the first months following ART initiation may be an important period during which continuity of care is consolidated or disrupted. Structuring this early period more intentionally, through focused review of missed visits, early response to interruption, and timely adjustment of service delivery intensity offers prevention of avoidable progression to prolonged disengagement. The observation that a proportion of men who were not active at 6 months subsequently re-engaged underscores that interruption is often reversible. Retention systems therefore benefit from maintaining accessible return pathways and reinforcing rapid follow-up within the first year of ART. Gaps in completion of the AHD package further highlight the importance of strengthening case-level implementation fidelity. Ensuring that each client receives the full complement of recommended AHD interventions remains central to improving both survival and continuity. Finally, although this study did not directly measure depressive disorders, qualitative narratives revealed psychosocial stressors including stigma, delayed disclosure, and challenges to masculine identity, that may influence early stabilization. A growing body of evidence demonstrates that mental health conditions are associated with poorer adherence and retention outcomes among people living with HIV (57). Integrating structured psychosocial assessment within early AHD care, with referral pathways for individuals requiring formal mental health support therefore represent an important complement to biomedical management. Future research should prioritise longer-term longitudinal designs (e.g., 3–5 years) to robustly evaluate sustained clinical outcomes, overall wellness trajectories, and patterns of long-term retention in care.

## Data Availability

The raw data supporting the conclusions of this article will be made available by the authors, without undue reservation.
